# Electron Scattering Cross Sections from Thiazole for Impact Energies Ranging from 1 to 1000 eV

**DOI:** 10.3390/molecules30051097

**Published:** 2025-02-27

**Authors:** Adrián García-Abenza, Ana I. Lozano, Juan C. Oller, Jaime Rosado, Francisco Blanco, Paulo Limão-Vieira, Gustavo García

**Affiliations:** 1 Instituto de Física Fundamental, Consejo Superior de Investigaciones Científicas, Serrano 113-bis, 28006 Madrid, Spain; agarciaa@aemet.es (A.G.-A.); alozano@irap.omp.eu (A.I.L.); 2 Agencia Estatal de Meteorología (AEMET), Centro Meteorológico de Málaga, Calle Demóstenes 4, 29010 Málaga, Spain; 3 Institut de Recherche en Astrophysique et Planétologie (IRAP), Université Toulouse, CNRS, CNES, Avenue du Colonel Roche, F-31028 Toulouse, France; 4 División de Tecnología e Investigación Científica, Centro de Investigaciones Energéticas, Medioambientales y Tecnológicas, 28040 Madrid, Spain; jc.oller@ciemat.es (J.C.O.); jrosadov@ucm.es (J.R.); 5 Departamento de Estructura de la Materia, Fısica Termica y Electronica eIPARCOS, Universidad Complutense de Madrid, 28040 Madrid, Spain; pacobr@ucm.es; 6 Laboratório de Colisões Atómicas e Moleculares, CEFITEC, Departamento de Física, Universidade NOVA de Lisboa, 2829-516 Caparica, Portugal; plimaovieira@fct.unl.pt; 7 Centre for Medical Radiation Physics, University of Wollongong, Wollongong, NSW 2522, Australia

**Keywords:** electron collisions, scattering cross sections, transmission experiments, electron scattering calculations

## Abstract

Total electron scattering cross sections (TCSs), in the energy range of 1–100 eV, have been measured with a high-resolution magnetically confined electron transmission apparatus, with total uncertainty limits estimated to be within ±5%. No previous experimental TCS data have been found for comparison. Electron attachment resonances, corresponding to transient negative ion formation, have been identified for energies below 20 eV by analyzing their contribution to the measured local maxima of the TCSs. Most of these resonances were observed for the first time. By means of our screening-corrected additivity rule (including interference effects) calculation method (IAM-SCAR + I), we extended TCS values to up to 1000 eV. This method also provides integral elastic, electronic excitation, and ionization cross sections for impact energies above 20 eV with total uncertainties of about ±10%. Comparisons, where possible, of the present electron scattering values with other values available in the literature are given.

## 1. Introduction

Thiazole (C_3_H_3_NS) is a five-membered aromatic heterocyclic compound which plays a major role in chemistry and biology [1,2]. Its unique heterocyclic structure containing both sulfur and nitrogen is part of many biologically active molecules [3], as is the case of penicillin [4], with many pharmacological applications ranging from antitumoral to antibacterial and antifungal. The biological relevance of thiazole is also clearly shown by its appearance in the structure of thiamine (vitamin B1) [5]. Moreover, in recent years, thiazole has been a subject of great interest in relevant scientific and technological areas such as astrochemistry, where it is a radioastronomy representative target of organosulfur chemistry [6], while for electronics, it may be used for the development of organic semiconductors [7] and to improve the efficiency of organic solar cells [8,9,10,11]. In addition, thiazole is a slightly prolate (κ = −0.166) asymmetric top with moderate and comparable dipole moment components along its two a- and b-principal axes (μ_a_ = 1.286 D; μ_b_ = 0.966 D) [12]. The precise structure [6] and electronic states [13,14] of the thiazole molecule have been widely studied. Moreover, photon interaction with thiazole has also attracted some attention, and studies on VUV photoabsorption [13], photoionization and photodissociation by strong laser fields [15], as well as photoelectron–photoion coincidence measurements [16] can be found in the literature.

However, despite the mentioned interest of thiazole in different fields, only a few studies have focused on electron interaction with this molecule. Modelli and Burrow [17] characterized temporary anion states of a whole set of heterocyclic compounds, including thiazole, by using an electron transmission spectroscopy technique. More recently, Li et al. [18] obtained a detailed dissociative electron attachment (DEA) fragmentation pattern by measuring the anion yield curves of thiazole and the other five-membered heterocyclic compounds as a function of the electron impact energy. From the theoretical perspective, Kossoski and Bettega [19] reported elastic integral and differential cross sections for electron scattering from five aza-derivatives of pyrrole, furan, and thiophene, including thiazole. In the case of thiophene, which has a similar structure to thiazole, Loupas et al. [20] studied the shape and core-excited resonances. Later on, Jani et al. [21] investigated the electronic states of thiazole and calculated electron scattering cross sections both for elastic and inelastic channels. In the surveyed literature, no experimental total electron scattering cross sections (TCSs) are currently available to the best of our knowledge.

The above considerations motivated the present study in which experimental TCSs for gaseous thiazole in the energy range of 1–100 eV were measured with a magnetically confined electron beam system [22]. In addition, total electron scattering, integral elastic, electronic-state excitation, and total ionization cross sections were calculated with our independent atom model with the screening-corrected additivity rule, including interference effects (IAM-SCAR + I) [23,24,25]. This method has been proven to be reliable within 10% for impact energies above 20 eV. Thus, here, we present the theoretical results for these elastic and inelastic channels in the energy range of 20–1000 eV.

The remainder of this paper is organized as follows: In Section 2, our experimental TCS results are presented, discussed, and compared with the available data in the literature, including a detailed analysis of the observed resonances. The experimental and theoretical methods used in this study are described in Section 3. Finally, some concluding remarks from this study are outlined in Section 4.

## 2. Results and Discussion

**Figure 1:**
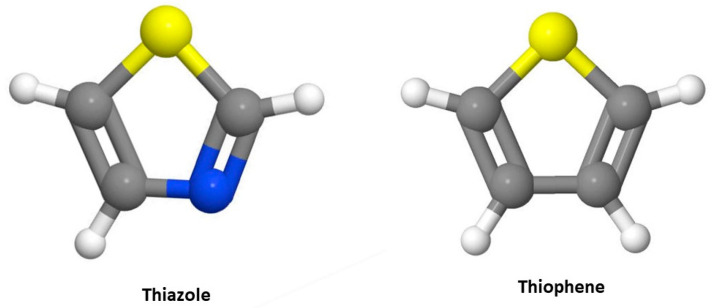
Molecular geometry and structure of thiazole (C_3_H_3_NS) and thiophene (C_4_H_4_S). ●, H; ●, C; ●, N; ●, S.

Thiazole is an azo-derivative of thiophene, and so comparing the present TCS values with our previous results for this molecule [26] is of great interest. The structure and geometry of both molecules are presented in Figure 1. In order to illustrate this process, Table 1 summarizes the main molecular properties [27,28,29,30] which are relevant to compare electron scattering cross sections.

**Figure 2:**
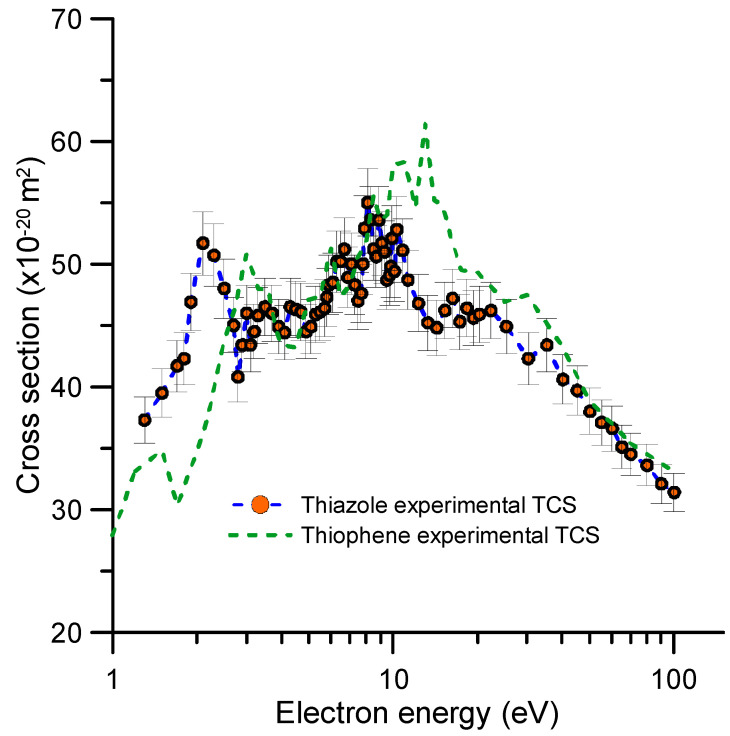
Experimental total electron scattering cross sections (TCS): **-**●**-**, thiazole; **---**, thiophene [26].

The experimental total electron scattering cross sections for gaseous thiazole are presented in Table 2 and plotted in Figure 2 along with our previous results for thiophene [26]. As this figure shows, the TCS magnitude and shape of both molecules are similar in the common energy range. This result may be expected by considering that both molecules have a very similar structure (see Figure 1) and the same number of electrons. The most notable differences are observed between 10 and 20 eV, where thiazole’s TCS values are lower than thiophene’s, and below 3 eV, where those of thiophene are lower than thiazole’s. The latter is probably due to the significant difference in their respective dipole moments (0.55 D thiophene [27] and 1.62 D thiazole [27]), which mainly affects lower energies. Although the difference in their respective molecular polarizabilities is much lower (see Table 1), it also acts in the same direction, increasing the thiazole TCS. As expected, the positions of their respective resonances appear different (a detailed description of thiophene’s resonances can be found in references [20,26,30]). The lower-lying resonance of thiophene appears at a higher energy than that of thiazole. This result agrees with previous studies [17,19] showing that the replacement of a CH group by a nitrogen atom leads to the displacement of the lower-lying π* resonances to lower energies. This behavior is related to the electron affinity of nitrogen [31], which is larger than that of the CH group [17,19]. As aforementioned, between 10 and 20 eV Thiophene’s TCS are higher in magnitude than those of thiazole, reaching a maximum difference of about 35% at 13 eV. This difference is probably associated with their respective ionization potentials (see Table 1). For higher energies, the TCS values of molecules tend to coincide. This result agrees with the semiempirical formulation of Garcia and Manero [32], which correlates the high-energy TCSs of several molecules with their respective numbers of electrons and molecular polarizabilities.

**Figure 3:**
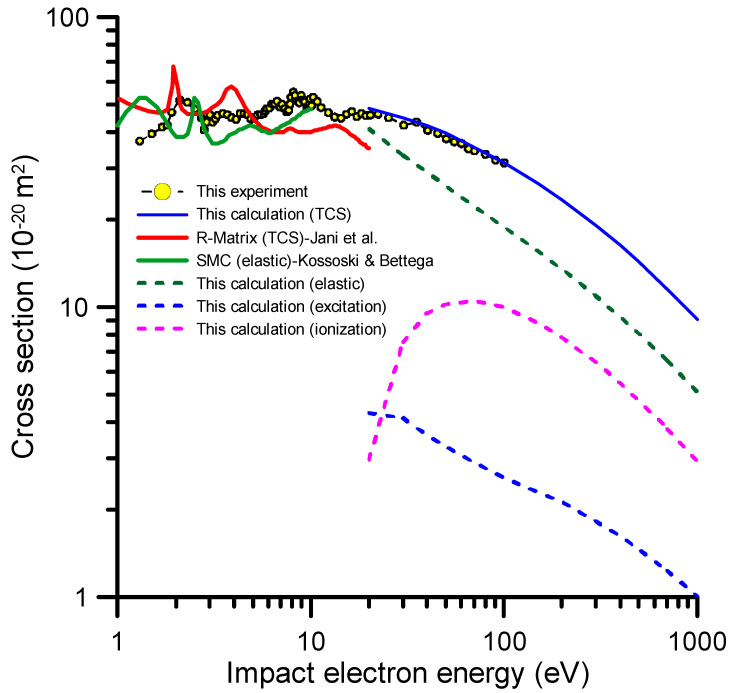
Experimental total electron scattering cross sections for thiazole molecule, together with our IAM-SCAR + I calculations and theoretical data from references [19] (**—**) and [21] (**—**). See legend in figure and text for further details.

As already mentioned, we applied our IAM-SCAR + I method (see Section 3.2) for impact energies ranging from 20 to 1000 eV to calculate elastic, electronic excitation, ionization, and total electron scattering cross sections with an estimated uncertainty of about 10%. The corresponding results are plotted in Figure 3, together with the experimental ones and other available theoretical results for lower collision energies. These were calculated by Kossoski and Bettega [19] and Jani et al. [21] using the Schwinger MultiChannel (SMC) method at the Static-Exchange-Polarization (SEP) level of approach and the R-matrix method with the Configuration Interaction (CI) scattering model, respectively. All the theoretical data shown in Figure 3 do not include dipole-Born corrections [30] as required by a fair comparison with the experimental data. Note that dipole interactions induce rotational excitations to the molecular target. The average rotational energy of thiazole at 300 K is about 1 meV, and the scattered electrons are strongly peaked in the forward direction; thus, due to energy resolution and acceptance angle limitations, the experimental TCS values do not account for rotational excitations. A close inspection of this figure shows that our theoretical experimental data agree very well in the overlapping energy range (20–100 eV). Since the accuracy of our calculation method increases with energy, we can use our theoretical data to accurately extrapolate the TCS values to up to 1000 eV. With respect to previous calculations, for lower collision energies, Kossoski and Bettega [19] reported integral elastic cross sections from thiazole for energies of up to 10 eV. However, except for the position of the resonances, there is overall good agreement between the experiment and calculation, and the theoretical magnitudes are systematically lower than the experimental ones above 3 eV. This discrepancy is expected, as this theoretical calculation does not account for inelastic channels in their scattering scheme. The R-matrix TCS values calculated by Jani et al. [21] agree better with the present experimental data as far as the position of the resonances is concerned. However, the absolute values of the cross section clearly disagree with ours, giving TCS values much lower in magnitude than the experimental data for energies above 3 eV. Note that these TCS values are even lower than the calculated elastic cross sections at these energies (3–20 eV). Finally, below 2 eV, experimental data tend to be lower in magnitude than the calculations. Two aspects may contribute to this disagreement, namely the systematic error due to the angular acceptance of the detector [22], which mainly affects lower energies by lowering the magnitude of the measured cross sections (see below in Section 3), and the approaches used by the different calculation methods to account for the dipole interaction, which tends to increase the magnitude of the calculated values, especially at lower collision energies [26].

Below 20 eV, different local maxima are discernible in the TCS profile. The peak position of each of them is presented in Table 2. These features would be likely related to the formation of transient negative ions (TNIs) via electron attachment (EA) processes. In order to analyze the position and shape of these resonances, we extracted the magnitude of the resonance peaks by subtracting from the TCS values the contribution of the background continuum, which is mainly due to elastic, vibrational, and electronic excitation processes. The result of this procedure is shown in Figure 3. A total uncertainty limit of ±11% was assigned to these results as a quadratic combination of the TCS and continuum background estimated uncertainties (5 and 10%, respectively).

**Figure 4:**
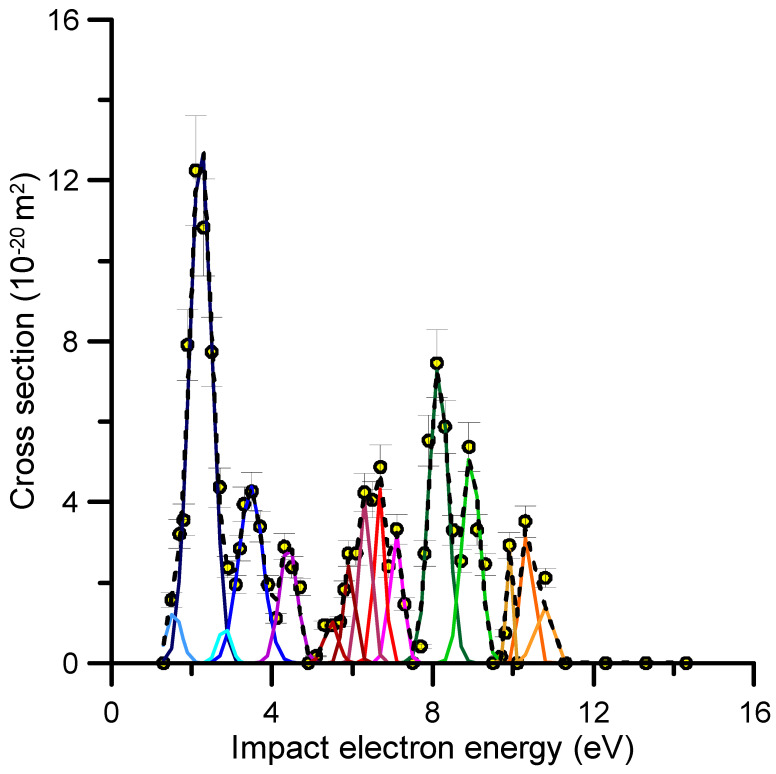
Resonance features extracted from the experimental TCS values in the energy range of 1–15 eV (see the text for details).

Figure 4 shows the gaussian fit of the resonance profiles derived from the experimental TCS values. Each peak was assumed to be originated by the temporary capture of an incident electron by the target molecule. The positions of these peaks are listed in Table 3 together with their respective electron attachment cross sections derived from the above subtraction procedure. By combining the TCS uncertainties (5%) with those assigned to the gaussian fitting procedure, an overall uncertainty limit of about 12% was estimated for these cross section values. The positions of the resonances observed and/or predicted by other authors are also included in Table 3.

The most notable feature in our measured TCSs peaks at 2.3 eV. This structure may correspond to the π_2_* anion state. Modelli and Burrow [17] measured a vertical attachment energy for this resonance of 2.27 eV, which is consistent with the present result. Regarding the theoretical outputs, the π_2_* resonance was located at 2.56 eV by Kossoski and Bettega [19] using the SEP approach and at 1.96 eV by Jani et al. [21] using the CI scattering model. Interesting to note is the complementary information about these low-energy resonances given by the most recent anion yield measurements obtained by Li et al. [18]. According to these authors, the fragment anions resulting from DEA to thiazole correspond to *m*/*z* 32 (S^−^), *m*/*z* 33 (SH^−^), *m*/*z* 50 (C_3_N^−^), and *m*/*z* 57 (C_2_HS^−^). As shown in Figure 8 in reference [18], all of the anion yield curves had a major peak with a maximum at 6.5 eV and a broad shoulder appearing at 4 eV for *m*/*z* 50, *m*/*z* 33, and *m*/*z* 32. Considering previous photo-absorption studies [13], Li et al. [18] concluded that all of these features can be considered as core-excited resonances. However, no significant anion yield signal was detected below 4 eV. The DEA-induced hydrogen loss fragment that they found at these low energies for isoxazole, methylisoxazole, and oxazole was not detected in the case of thiazole. However, as shown in Figure 3, the most intense resonance we found on the TCS was placed at 2.3 eV. We can then suggest that this resonance decays mainly via electron detachment, leading to neutral reaction products. Concerning the resonance we found at 3.5 eV, a similar local maximum of the TCS value around 3.3 eV was also observed for thiophene [26] (see Figure 2). Muftakhov et al. [33] attributed it to a Feshbach resonance whose parent state would be the first excited triplet of thiophene. This could also apply for thiazole considering that its first excited triplet has a vertical excitation energy of 3.8 eV [13]. Kossoski and Bettega [19] found a σ_ring_* resonance at 4.5 eV. Thus, we could assign the structure observed at 4.5 eV to the formation of this anion state. Above 5 eV, different features are observed in our TCS profile. However, the characterization of those structures is more challenging as they can be related to one or more core-excited resonances, which would not be well resolved by our experiment due to energy resolution limitations, thus remaining open to further studies. Nonetheless, the ion yield measurements of Li et al. [18] reported broad structures around 6.5 eV for all anion fragments and at 8.7 eV for the formation of *m*/*z* 57 and *m*/*z* 32. These results perfectly agree with the positions of the resonances derived from our TCS measurements. Our resonances at 5.5, 5.9, 6.3, and 6.7 eV may contribute to the former anion structure, and those at 8.1 and 8.9 eV to the latter. Similarly, the resonances around 10 eV could contribute to the continuum anion yield signal detected for *m*/*z* 57 and *m*/*z* 32 in reference [18] for impact energies above 10 eV. Note that these energies are above the ionization energy of thiazole (9.5 eV), and non-resonant dipolar dissociations compete with the DEA channel. Resonances at 1.5, 2.9, and 10.8 eV were tentatively considered just to optimize the fitting curve to the experimental data, but their physical entity cannot be confirmed within the uncertainty limits of this experiment.

## 3. Materials and Methods

### 3.1. Magnetically Confined Electron Beam (MCEB) System

The present measurements of the TCSs for electron scattering from thiazole molecules were performed in an experimental setup described in detail elsewhere [22]; thus, only relevant details are discussed here. The entire system is surrounded by an intense (0.2 T) axial magnetic field which confines and guides the electron beam all along its constituent chambers. Briefly, the primary beam was obtained by the thermionic emission of a hairpin filament with an initial energy spread of around 500 meV. Then, it was extracted into a nitrogen gas trap, where electrons were maintained at around 50 ms by applying a pulsed electric potential barrier at both ends of this chamber. During this time, the primary electron beam was cooled down by successive collisions with the nitrogen molecules inside this chamber. With this procedure, as described in detail in Ref. [22], the initial energy spread of the electron beam was reduced to around 100 meV. The electron beam was subsequently transported to the scattering chamber, where the target molecule was admitted via a leak valve. The gas pressure inside this chamber was kept constant and measured using a Baratron capacitance manometer. Finally, prior to their detection by a double microchannel plate (MCP) electron multiplier operating in single counting mode, electrons were energy-analyzed by a retarding potential analyzer (RPA). By choosing the operating point, as indicated in Ref. [22], the effective energy resolution could be substantially improved, reducing to about 50 meV. Measurement conditions, as well as data acquisition and analysis, were controlled by custom-designed LabView (National Instruments, Austin, TX, USA) programs.

The total electron scattering cross sections were obtained for each energy by using the well-known Beer–Lambert attenuation law and assuming an ideal gas behavior. In addition, to avoid multiple scattering processes and assure the validity of the Beer–Lambert law, a convenient range of gas pressures was determined and maintained during the attenuation measurements. To reduce statistical uncertainties to about 4%, at least five attenuation curves were measured for each impact energy. By considering all the noticed random error contributions (see Ref. [22] for details), a total uncertainty limit of ±5% was obtained for the present measurements. Basically, this total ±5% assigned to the absolute TCS values was the result of adding in quadrature the statistical uncertainties (4%), absolute pressure and temperature determination (1%), and fitting procedures (1%).

Finally, it is important to note that, due to the magnetic confinement, in this experimental setup, the angular resolution was linked to the energy resolution and the incident electron energy (see Ref. [22] for further details). In these conditions, the missing angle contribution increases for decreasing energies. This tends to lower the measured TCS from the actual value, especially for lower collision energies. In addition, for polar molecules, as is the case for thiazole, this effect will increase since rotational excitations strongly peak in the forward direction, especially at lower energies. The present results were not corrected for this systematic error. Consequently, in order to make a fair comparison with other experimental and/or theoretical data, the experimental angular acceptance should be considered. These considerations are especially critical when we discuss rotational excitation processes. Transmission experiments with energy resolutions of the order of the present one are not able to account for rotational excitations (the average rotational excitation energy of thiazole is much lower than 1 meV), while some calculations indirectly include rotational excitation via dipole-Born corrections [23].

### 3.2. Theoretical Calculations

Differential and integral elastic cross sections as well as integral inelastic electronic excitation and ionization cross sections were obtained through our IAM-SCAR+I method described in detail elsewhere [23,24,25]. In short, the molecular target was considered as an aggregate of its individual atoms, where each atom is represented by an ab initio optical potential. The real part of the potential accounts for elastic scattering, while the imaginary part represents the inelastic processes, i.e., the “absorption part”. The differential scattering cross sections were obtained from the atomic data by the screening corrected additivity rule (SCAR) procedure, incorporating interference (I) corrections by summing all of the atomic amplitudes, where the phase coefficients are included. Then, by integrating all of the scattered angular ranges, the integral scattering cross sections (ICSs) were obtained. The rotational excitation cross sections stem from the first-Born approximation. Finally, the calculated TCS was obtained by summing up the elastic, electronic excitation, and ionization cross sections. This calculation procedure has been applied to a great number of molecular targets from diatomic to polyatomic molecules (see Refs. [23,24,25,26] and references therein). The uncertainties assigned to the present calculated data were derived from comparisons with accurate experimental values. Probably the most representative molecular target for this comparison is tetrahydrofurane (THF). For this molecule, we measured, calculated, and compared values with available data to establish a complete cross section set [34] which was validated by electron transport simulations [35] and machine learning procedures [36]. We could then safely assign to the present elastic, electronic excitation, ionization, and total electron scattering cross sections an overall uncertainty limit of ±10% for electron impact energies ranging from 20 to 1000 eV.

## 4. Conclusions

Experimental electron scattering total cross sections for thiazole in the energy range of 1–100 eV were obtained for the first time from a magnetically confined electron transmission beam system. Additionally, these TCSs served to investigate the accuracy of three ab initio electron scattering calculation methods: the IAM-SCAR + I method for intermediate and higher energies, and the SMC and R-matrix methods suitable for lower energies. An overall good agreement was found between the experiment and the calculated IAM-SCAR + I and SMC results, while for those obtained by Jani et al. [21], important discrepancies were noted. On the other hand, below 20 eV, many features related to the formation of TNI were identified in the TCS profile, some of them for the first time. In particular, the position of the well-known lower-lying resonance π_2_* was found to be in good agreement with both previous experimental and theoretical results. The features observed above 5 eV in the experimental TCSs are consistent with previous DEA experiments, allowing us to identify some electron attachment resonances with the formation of different anion fragments. In some cases, electron detachment processes were identified as the main decay channels. Further studies will be needed to characterize these resonances.

Finally, the present results will serve to further assess a complete self-consistent electron scattering process from a gaseous thiazole cross section dataset as required in modeling electron transport applications.

## Figures and Tables

**Table 1:** Relevant molecular properties of thiazole and thiophene for cross section comparisons.

Molecular Property	Thiazole	Thiophene
Dipole moment (D)	1.62 [27]	0.55 [27]
Number of electrons	44	44
Molecular polarizability (Å^3^)	8.43 [28]	9.27 [28]
Optical electronic excitation threshold (eV)	5.4 [13]	5.0 [29]
Ionization energy threshold (eV)	9.5 [27]	8.86 [27]

**Table 2:** Experimental total electron scattering cross sections for thiazole in SI units in present study. Total uncertainty limits are within 5% (see text for details).

Energy (eV)	TCS (10^−20^ m^2^)	Energy (eV)	TCS (10^−20^ m^2^)
1.3	37.3	7.9	52.9
1.5	39.5	8.1	55.0
1.7	41.7	8.3	53.6
1.8	42.3	8.5	51.2
1.9	46.9	8.7	50.6
2.1	51.7	8.9	53.6
2.3	50.7	9.1	51.7
2.5	48.0	9.3	51.0
2.7	45.0	9.5	48.7
2.8	40.8	9.7	49.1
2.9	43.2	9.8	49.8
3.0	46.0	9.9	52.1
3.1	43.4	10.1	49.4
3.2	44.5	10.3	52.8
3.3	45.8	10.8	51.1
3.5	46.5	11.3	48.7
3.7	46.0	12.3	46.8
3.9	44.9	13.3	45.2
4.1	44.4	14.3	44.8
4.3	46.5	15.3	46.2
4.5	46.3	16.3	47.2
4.7	46.1	17.3	45.3
4.9	44.5	18.3	46.4
5.1	44.9	19.3	45.6
5.3	45.9	20.3	45.9
5.5	46.1	22.3	46.2
5.7	46.4	25.3	44.9
5.8	47.3	30.3	42.3
5.9	48.3	35.3	43.4
6.1	48.5	40.3	40.6
6.3	50.2	45.3	39.7
6.5	50.2	50.3	38.0
6.7	51.2	55.3	37.1
6.9	48.9	60.3	36.6
7.1	50.0	65.3	35.1
7.3	48.3	70.3	34.5
7.5	47.0	80.3	33.6
7.7	47.6	90.3	32.1
7.8	50.0	100.3	31.4

**Table 3:** The positions of the resonance peaks (in units of eV) observed in our experiment (see also Figure 3) with their assigned electron attachment cross sections with total uncertainty limits of about 12%. Experimental resonance energies derived from the transmission spectra of Modelli and Burrow [17] and the anion current measurements of Li et al. [18] are also shown together with the theoretical energy positions calculated by Kossoski and Bettega [19] using the SMC-SEP method and by Jani et al. [21] (R-matrix-CI method).

Resonance	Peak Position (eV)	Electron Attachment Cross Section (10^−20^ m^2^)	Other Experimental Positions (eV)	Other Calculated Positions (eV)
$\sigma_{sc}^{*}$	1.5	1.20	1.6 [17]	
$\pi_{2}^{*}$	2.3	12.7	2.27 [17]	2.56 [19] 1.96 [21]
	2.9	0.814		
	3.5	4.37		
$\sigma_{ring}^{*}$	4.5	2.80		4.5 [19]
	5.5	1.01		
	5.9	2.42		
	6.3	4.06		
	6.7	4.71		
	7.1	3.21		
	8.1	7.27		
	8.9	5.09		
	9.9	2.81		
	10.3	3.33		
	10.8	1.38		

## Data Availability

The data presented in this study are available in the article.
